# Integrative Analysis of HTNV Glycoprotein Derived MHC II Epitopes by *In Silico* Prediction and Experimental Validation

**DOI:** 10.3389/fcimb.2021.671694

**Published:** 2021-07-19

**Authors:** Hao Sun, Zhenhua Lu, Guoyun Xuan, Ning Liu, Tianhu Wang, Yang Liu, Mingfu Lan, Jiahao Xu, Yuancai Feng, Shuang Xu, Yuchen Lu, Baozeng Sun, Jinpeng Zhang, Xiyang Zhang, Yuanjie Sun, Shuya Yang, Yun Zhang, Yusi Zhang, Linfeng Cheng, Dongbo Jiang, Kun Yang

**Affiliations:** ^1^ Department of Immunology, Basic Medicine School, Air-Force Medical University (The Fourth Military Medical University), Xi’an, China; ^2^ Department of Epidemiology, Public Health School, Air-Force Medical University (The Fourth Military Medical University), Xi’an, China; ^3^ Department of Surgery, Jinling Hospital, Nanjing, China; ^4^ Department of Microbiology, Basic Medicine School, Air-Force Medical University (The Fourth Military Medical University), Xi’an, China

**Keywords:** Hantaan virus glycoprotein, major histocompatibility complex II epitope, *in silico* prediction, enzyme-linked immunospot assay, pan-major histocompatibility complex reaction

## Abstract

Hantaan virus (HTNV), the causative pathogen of hemorrhagic fever with renal syndrome (HFRS), is a negative RNA virus belonging to the Orthohantaviridae family. HTNV envelope glycoprotein (GP), encoded by the genomic medium segment, is immunogenic and is therefore a promising vaccine candidate. Major histocompatibility complex class I (MHC-I) epitopes derived from HTNV has been extensively studied, but little is known of MHC-II epitopes. In silico predictions based on four databases indicated that the full-length HTNV GP has 1121 15-mer epitopes, of which 289 had a high score for binding to the human and murine MHC-II superfamily. It found that epitope ILTVLKFIANIFHTS could potentially bind most MHC-II molecules covering human and murine haplotypes. Dominant epitopes were validated by enzyme-linked immunospot assay of splenocytes from immunized mice; 6 of 10 epitopes supported the predictions including TATYSIVGPANAKVP, TKTLVIGQCIYTITS, FSLLPGVAHSIAVEL, CETYKELKAHGVSCP, CGLYLDRLKPVGSAY, and NLGENPCKIGLQTSS. Conservation analysis of dominant epitopes revealed host–virus interactions without geographic stratification, thus meeting the requirements of candidate vaccines for large-population prophylaxis. These findings provide insight into hantavirus antigenicity and suggest that vaccines targeting MHC-II could provide immune protection in large population to complement symptomatic therapies for the treatment of HFRS.

## Introduction

Hantaviruses belong to the Bunyavirales order, Hantaviridae family, and *Orthohantavirus* genus (Shimizu et al., 2018) and are divided into Old and New World groups. The former includes Hantaan virus (HTNV), Seoul virus, Dobrava-Belgrade virus, and Puumala virus, which cause hemorrhagic fever with renal syndrome (HFRS); and the latter includes Andes virus and Sin Nombre virus, which are the major causative pathogens of hantavirus pulmonary syndrome (HPS). HFRS is characterized by renal dysfunction and hemorrhage and HPS by acute respiratory failure. Both diseases have a high mortality rate.

Hantavirus has a tripartite genome with small, medium, and large segments encoding nucleocapsid protein, envelope glycoprotein (GP), and viral RNA polymerase, respectively (Li et al., 2017). The pathogenesis of HFRS involves immune complex activation, complement activation, T- and B-cell responses, and HTNV-induced cytokine production (Jiang et al., 2016). GPs, which include Gn and Gc, are the main origin of neutralizing antibody. Helper T cells produce cytokines that promote antibody production and isotype switching by B cells and facilitate the differentiation of CD8 + T cells into memory cells (Sadegh-Nasseri, 2016). Major histocompatibility complex class II (MHC-II) and peptides derived from antigen form peptide (p)MHC-II complexes for antigen presentation to CD4+ T cells. Thus, both humoral and cellular immunity are activated by CD4, which plays an important role in infection and immunity.

MHC-II proteins on antigen-presenting cells (APCs), such as dendritic cells, macrophages, and B cells, bind and display peptides from self and foreign proteins at the cell surface that interact with CD4+ T cells. Most peptides bound by MHC-II proteins are 13 to 25 amino acid (a.a.) residues, with an average length of 15 residues (Painter and Stern, 2012). MHC-II consists of α and β chains that form a noncovalent bond; bound peptides freely extend outside the complex but contain a stretch of residues that directly interact with the binding groove of the complex (Germain, 1995). CD4+ T cells, which are critical for cellular, humoral, and long-term immune response, are effectively primed after antigen processing by MHC-II in HTNV immunity (Kaech et al., 2002; Janssen et al., 2003; Shedlock and Shen, 2003; Rocha and Tanchot, 2004; Khanolkar et al., 2007; Smith et al., 2008). The aim of this study was to identify specific epitopes recognized by MHC-II, which have not been previously reported and aid in vaccine design.

## Materials and Methods

### Amino Acid Sequence Retrieval

The glycoprotein (GP, accession no: Y00386.1) of Hantaan virus 76-118 were obtained from NCBI GenBank and used as an input for various bioinformatics tools for epitope prediction, conservation analysis, and the experiment. For calculating the conservation rank of predicted epitopes overlapping HTNV GP among Hantaan virus strains, the protein sequences of different isolates (148 envelope glycoproteins as shown in **Supplementary Table 1**) were obtained from NCBI GenBank.

### Epitope Prediction

For H2-A and H2-E epitope prediction, Web-based tools like IEDB recommended method (http://tools.iedb.org/mhcii/), NetMHCIIpan 3.2 Server (http://www.cbs.dtu.dk/services/NetMHCIIpan/) (Jensen et al., 2018), Rankpep (http://imed.med.ucm.es/Tools/rankpep.html) (Reche et al., 2002; Reche et al., 2004; Reche and Reinherz, 2007), and SYFPEITHI (http://www.syfpeithi.de/bin/MHCServer.dll/EpitopePrediction.htm) were used to predict. Predicted epitopes are chosen, which are highlighted in red for Rankpep or ranking top 2 percentile for IEDB and NetMHCIIpan and top 2% of number for SYFPEITHI. Finally, the epitopes predicted by any tools are regarded the dominant epitope.

The epitopes of alleles of major human leukocyte antigen (HLA)-II supertypes like DRB1 (DRB1*0101, DRB1*0301, DRB1*0401, DRB1*0405, DRB1*0701, DRB1*0802, DRB1*0901, DRB1*1101, DRB1*1201, DRB1*1302, DRB1*1501), DRB3/4/5 (DRB3*0101, DRB3*0202, DRB4*0101, DRB5*0101), DQA1/DQB1 (DQA1*0501/DQB1*0201, DQA1*0501/DQB1*0301, DQA1*0301/DQB1*0302, DQA1*0401/DQB1*0402, DQA1*0101/DQB1*0501, DQA1*0102/DQB1*0602), DPB1 (DPB1*0101, DPB1*0201, DPB1*0401, DPB1*0402, DPB1*0501, DPB1*1401) were predicted using four bioinformatics tools include IEDB recommended, NetMHCIIpan, Rankpep, and SYFPEITHI. It is estimated that these alleles cover more than 98% in all major ethnicities worldwide (Greenbaum et al., 2011). Eventually, we chose the predicted epitopes which are top 2 percentile rank for IEDB recommended and NetMHCIIpan, highlighted in red for Rankpep and top 2% of number for SYFPEITHI. The epitopes which were predicted by more than two prediction tools were subjected to the sequential studies.

### Viruses and the Inactivated Vaccine

HTNV 76-118 strains were propagated in BHK-21 cells (Jiang et al., 2017). The bivalent HFRS inactivated vaccine (HANPUWEI^®^) which was produced by the Changchun Institute of Biological Products Co., Ltd., Changchun, China was used in this study. The vaccine was made of inactivated and purified HTNV and SEOV in Hamster Kidney Cells (there is no monovalent HFRS vaccine available in China).

### Animals and Immunization

Eight-week-old specific-pathogen-free female mice of four kinds were purchased from the Laboratory Animal Centre of the Fourth Military Medical University. Three types of inbred mice included c57, BALB/c, and C3H within MHC-II haplotypes of H2b, H2d, and H2k, respectively, and the other one was closed colony mice of Kunming. They were randomly divided into two groups of each kind and 10 mice in each group. The first groups of mice were inoculated with 50 μl 1:5 diluted bivalent inactivated vaccine (referring to the instructions of the manufacturer to determine the immune dose). The vaccine is injected every 3 weeks, twice in total. The mice in other groups were injected with 50 μl 100 TCID50 diluted HTNV strain 76-118 only once. Five weeks after the first immunization, the immunized mice were sacrificed. ELISpot assay was used for cellular evaluation. All animals were kept in a pathogen-free animal laboratory with quiet raising condition. The animals were fed with standard feeds and filtered water and bedding was changed every week. More than 10 h of continuous night time were ensured to maintain circadian rhythm. Sterile gloves were taken in all experimental operations. The sacrifice of animals was inhaled carbon dioxide. The whole procedures for care and use of animals were approved by the Ethics Committee of the Fourth Military Medical University’s Animal Center and all applicable institutional and governmental regulations concerning the ethical use of animals were followed.

### Peptides and ELISpot Assay

Full-length HTNV GP yielded 147 peptides with an 8-amino acid overlapping 15-mer design strategy, including 83 peptides of Gn and 64 peptides of Gc (ChinaPeptides, Shanghai, China) because of the inherent difficulties in their production. As mentioned previously, single peptides were diluted with 20 μg/ml in PBS for INF-γ ELISpot assay (Jiang et al., 2015; Jiang et al., 2017). Briefly, IFN-γ–specific capture antibody was diluted with 5 μg/ml (1:250) in sterile PBS and coated ELISpot plates overnight at 4°C. Mice were sacrificed, and their spleens were dissected, and the monocyte suspension was ground. After erythrocyte lysis, the splenocytes were washed and re-suspended. Two hours after the ELISpot plates were blocked with RPMI-1640 containing 10% fetal bovine serum at room temperature, 1 × 10^6^ splenocytes were added to each pore and stimulated with a final concentration of 20 μg/ml synthetic GP peptides. The plates were cultured in a 5% CO_2_ incubator of 37°C for 24 h. Completed medium was used as the negative control. Con A (10 μg/ml) was used as the positive control. After incubation, the culture plates were washed with H_2_O and PBST, and then incubated with 2 mg/ml relevant biotinylated rat anti-mouse IFN-γ antibody at room temperature for 2 h. After washing with PBST, the plates were incubated with streptavidin-HRP 1:100 for 1 h, and then 3-amino-9-ethylcarbazole (AEC; BD Pharmingen) was added to the HRP substrate, and the reaction was stopped by washing with water. After air-drying, use the AID ELISpot Reader Classic–ELR06 (AID, Strassberg, Germany) to count IFN-spots generated by AEC substrate (BD Pharmingen). Each experiment was performed in triplicate, and all the results were shown as the average value of spot forming cells (SFC) per 10^6^ splenocytes.

### Experimental Results Analysis

The criterion for dominant epitopes verification corresponded to the first quarter of values from high to low by ELISpot assay. To compare the experimental and predicted epitopes, predicted epitopes were clustered to eight-amino acid overlapping 15-mer strategies parallel to experimental peptides and the predicted epitopes with common part more than 12 amino acids were clustered. The three groups of epitopes in forecast, inactivated and live virus were drawn into Venn diagram with VENNY 2.1 (https://bioinfogp.cnb.csic.es/tools/venny/index.html).

### Conservation Analysis

One hundred and forty-eight protein sequences of different isolates were used for following analysis. ClustalW program was utilized in MEGA X software suite with default settings to carry out multiple sequence alignments of GP of Hantaan virus strains (Kumar et al., 2018). We displayed the conservation level in multiple sequence alignment by WEBLOGO 3.0 (Crooks et al., 2004), and the predicted epitopes were underlined in blue, red, and orange for predicted epitopes from human, mouse, and both, respectively. Furthermore, we entered these selected epitope sequences into BLASTP program and calculated the status of conservation among protein sequences of Hantaan virus (Taxid:11599), other hantaviridae (Taxid:1980413), human (Taxid:9606), and mouse (Taxid:10088). The epitope was regarded within high conservation when e-value was lower than 10^−5^. The epitopes which were conserved between human and mouse were excluded. **Supplementary Table 4** shortlisted the epitopes which are conserved in Hantaan virus strains as well as in other hantaviridae.

### The Interactions Between MHC-II and Epitopes

The percentile rank of binding strength obtaining from NetMHCIIpan profiled the interaction between MHC-II and epitopes. Prism 8.0.1 was used to draw a heatmap with 35 MHC-II subtypes and 1,121 epitopes. Heatmap judged the potentials of MHC-II presenting GP and GP being presented. It illustrated similarities and differences in immune responses to GP among MHC-II superfamilies and alleles of human and mice. The structure of GP activating CD4+T cells was featured “hot-zone” for vaccine design.

The pheatmap provides more control over dimensions and appearance, and allows using k means clustering to aggregate rows. The data were processed by normalization. The pheatmap package (Version 1.0.12) was used for the bidirectional hierarchical clustering, and the expression values were presented by heatmaps (Cheng and Wang, 2020).

### The Geographical Stratification of MHC-II Distribution

The web (http://www.allelefrequencies.net/hla6006a.asp) was used to obtain the geographical distribution of different HLA-II subtypes and the results of heatmaps were determined whether there were bias in virus immunity among individuals caused by geographical regions and ethnic groups.

### Statistical Analysis

All the data for each group are expressed as means ± SDs. Unpaired t-test was performed to evaluate the differences between the groups. The analyses were conducted using GraphPad Prism version 5.0. P-value < 0.05 was considered statistically significant. All the statistical operations were two-tailed analyses.

## Results

### *In Silico* Analysis Obtained Potential MHC-II Epitopes

In order to predict potential MHC-II epitopes in HTNV envelope GPs with different binding affinities, we performed a bioinformatic analysis for mouse MHC-II sequences (H2-A and H2-E) and alleles of major HLA-II supertypes. **Table 1** lists the potential epitopes obtained with each of the prediction tools. There were 181 epitopes in H2 and 148 in HLA-II, of which 37 were duplicates; thus, there were 289 unique epitopes (**Supplementary Table S2**). The highest number of H2 epitopes were for H2-Ad (51 peptides of full-length GPs), whereas DRB1 epitopes were the most highly represented among HLA-II subtypes (134 peptides of full-length GPs).

**Table 1:** HTNV glycoprotein MHC-II epitope prediction.

MHC-II Haplotypes	Prediction tools	Glycoprotein peptide hits	Glycoprotein (Short listed)
	IEDB-recommended	289	
DRB1	NetMHCIIpan	72	134
	Rankpep	118	
	SYFPEITHI	101	
	IEDB-recommended	133	
DRB3/4/5	NetMHCIIpan	29	18
	Rankpep	0	
	IEDB-recommended	71	
DQA1/DQB1	NetMHCIIpan	26	7
	Rankpep	6	
	IEDB-recommended	17	
DPB1	NetMHCIIpan	13	3
	IEDB-recommended	8+4 (b,d)	
H2-A	NetMHCIIpan	8+2+5+10+5+8 (b,d,k,q,su)	124
	Rankpep	27+22+7+0+7+0 (b,d,k,q,su)	
	SYFPEITHI	20+20 (d,k)	
	IEDB-recommended	9 (d)	
H2-E	NetMHCIIpan	6+3 (d,k)	63
	Rankpep	0+9+8+2 (b,d,k,s)	
	SYFPEITHI	20+20 (d,k)	

The right column shows the number of epitopes of mouse MHC-II (H2-A, H2-E) and some alleles of major HLA-II supertypes (DRB1, DRB3/4/5, DQA1/DQB1, DPB1) predicted using indicated prediction tools based on Hantaan glycoprotein sequences.

HTNV, Hantaan virus; IEDB, immune epitope database; MHC, major histocompatibility complex.

### ELISpot Assay Verified Predicted Epitopes

**Figure 1:**
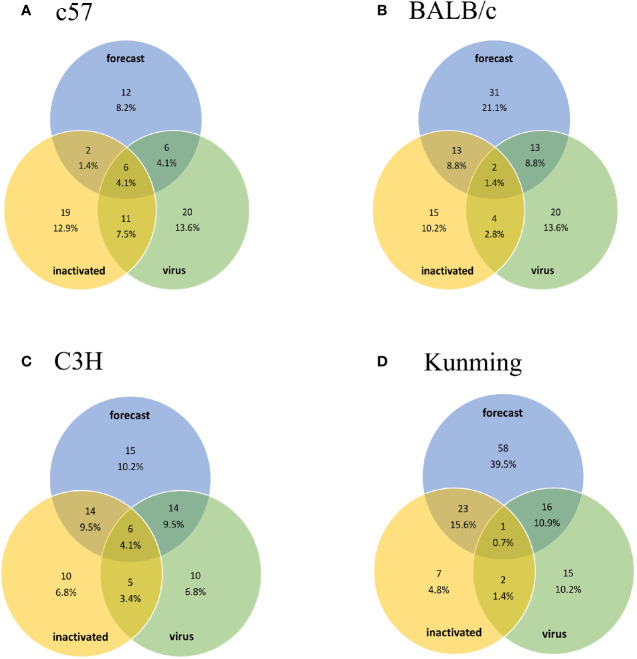
Venn diagram of predicted epitopes and epitopes obtained experimentally using inactivated HTNV vaccine and live virus. The number of overlapping epitopes and their proportions are shown. Groups **(A–D)** are the results obtained from C57, BALB/c, C3H, and Kunming mice, respectively. The percentage represents the ratio of the number of epitopes to the total number of experimentally determined epitopes (147 peptides including 83 Gn and 64 Gc peptides).

**Figure 2:**
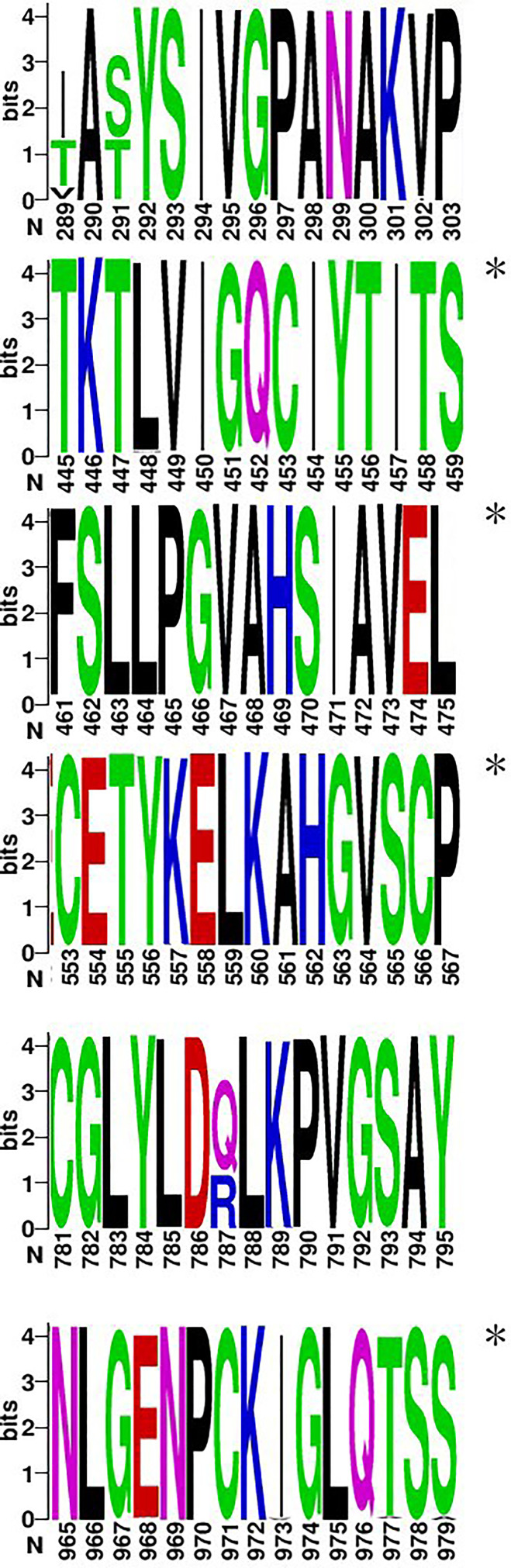
Six epitopes selected from hotspots and clusters (a.a. 289–303, 445–459, 461–475, 553–567, 781–795, and 965–979). The figure was generated from the conservation analysis carried out using WEBLOGO. The height of symbols within the stack indicates the relative frequency of each a.a. at that position, with a.a.s colored according to their chemical properties as follows: polar (G, S, T, Y, C, Q, and N), green; basic (K, R, and H), blue; acidic (D and E), red; and hydrophobic (A, V, L, I, P, W, F, and M), black. Highly conserved epitopes are indicated by an asterisk (*).

Injection of vaccine or live virus induced cellular responses specific to HTNV. In C57, BALB/c, C3H, and Kunming mice, 38, 34, 35, and 33 epitopes, respectively, were obtained by vaccine injection and 43, 39, 35, and 34, respectively, were obtained by live virus injection (**Supplementary Figure S1**). The dominant epitopes obtained in previous studies are listed in **Supplementary Table S3** (Ma et al., 2015). These results were compared with predicted epitopes (**Figure 1**) in a Venn diagram analysis of 147 15-mer peptides. The predicted number of epitopes was greater than the number obtained experimentally, with some overlaps and differences between experimental datasets. Nonetheless, most of the predicted epitopes were verified in experiments with the ELISpot assay, except in Kunming mice; as these mice are a closed colony with a relatively complex genetic background, they are expected to have a greater number of predicted epitopes than the other 3 homozygotic lines. There were 10 hotspots associated with the human or mouse immune responses, of which 6 (3 in each species) were consistent with predictions (**Figure 2**). For the image drawn by WEBLOGO 3.0, height of symbols within the stack indicates the relative frequency of each a.a. at that position. The highly conserved epitopes that marked with asterisks are the peptides with specific amino acid at every single position. These results confirm the accuracy of the *in silico* analysis and effectiveness of ELISpot validation of the predicted epitopes.

### HTNV Epitopes Are Highly Conserved in Hantaan Virus and Orthohantavirus

To evaluate the conservation of qualified epitopes among HTNV GPs, we performed multiple sequence alignment of 148 HTNV strains. The dominant HTNV epitopes showed high intraspecific conservation and similar peptides location within GP protein sequences (**Supplementary Figure S2**). The epitopes for MHC-II obtained by BLASTP analysis were classified into 2 categories—namely, HTNV− and HTNV+ (**Supplementary Table S4**). All were found to be conserved in Orthohantavirus. Some epitopes were conserved in some HTNV strains but not in others, possibly reflecting adaptation to local environments. None of the epitopes showed homology to human or mouse protein sequences.

### Regionally Distributed Epitopes and MHC-II With Similar Immune Capacities

We examined the binding strength between epitopes and MHC-II subtypes (**Supplementary Figure S3**). MHC-II includes H2 (H2-A and H2-E) and major HLA-II supertypes (DRB1, DRB3/4/5, DQA1/DQB1, and DPB1). The GP of HTNV strain 76-118 is composed of 1135 amino acids (a.a.), and the epitope was located from a.a. 1–1121. In general, the binding strength of epitopes showed regional distribution; epitopes with strong binding affinity in the majority of subtypes were located at a.a. 1–18, 138–175, 208–210, 438–507, 779–800, and 910–927, whereas those at 752–777 and 1022–1047 showed poor binding affinity. Allele DR, DQ, and DP have a similarity in affinity with peptides. Epitopes are clustered in distribution, and DQ is more scattered in location. H2 had broader distributions than a single HLA-II supertype; thus, the rank order of the overall distribution of different MHC-II subtypes was H2 > DQA1/DQB1 and DPB1 > DRB1 and DRB3/4/5. On the other hand, HLA-II supertypes showed greater overall coverage of GP-derived epitopes than H2 alleles, which had a rank order of DRB1 and DRB3/4/5 > DQA1/DQB1 and DPB1 > H2. The narrower distribution of MHC-II subtypes was associated with a higher concentration of the corresponding epitopes.

**Figure 3:**
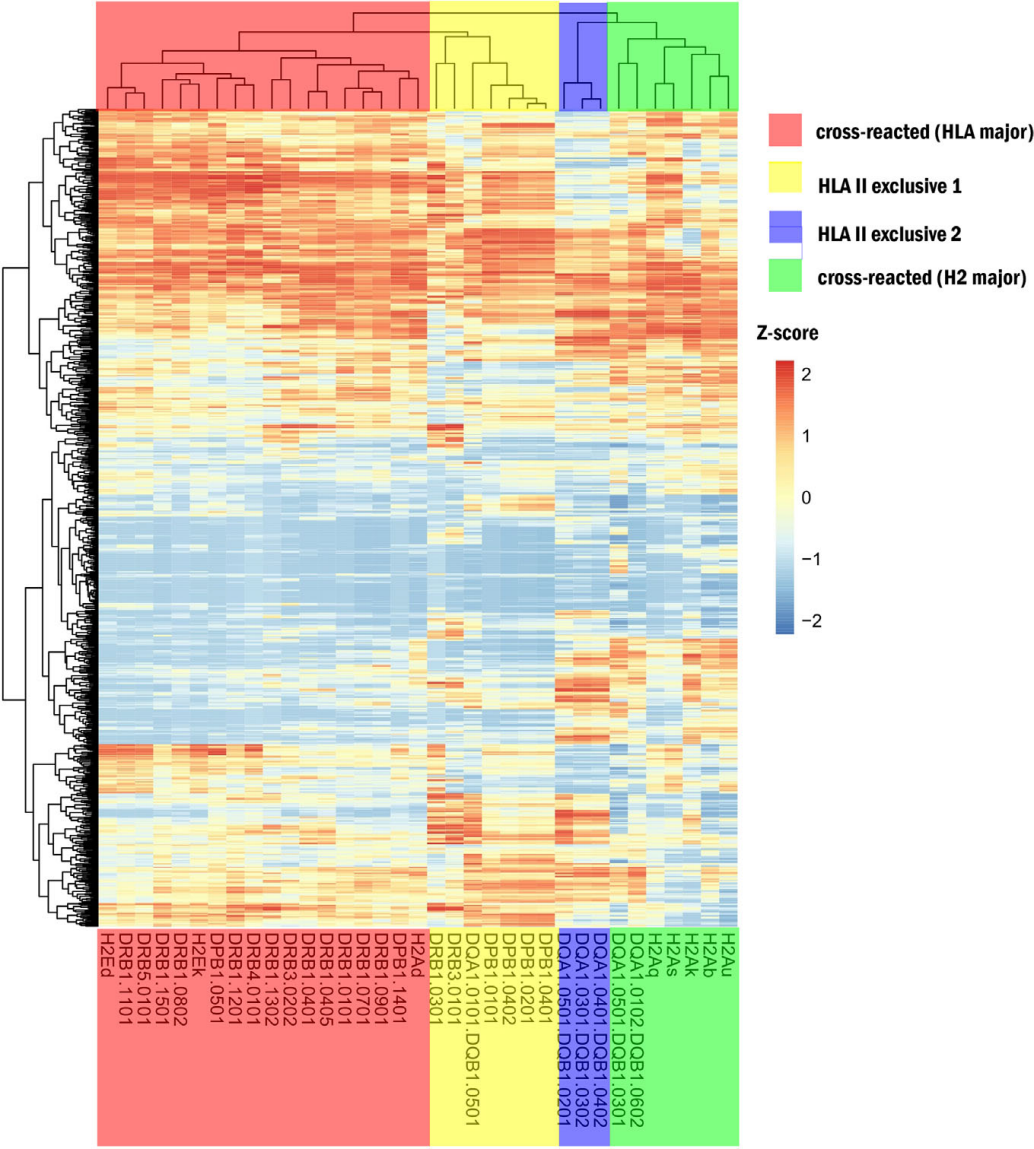
Heatmap for different MHC-II. The red region shows cross-reactive epitope clusters (HLA major) including DRB1 and 3 H2 subtypes (H2-Ed, k, and H2-Ad) corresponding to BALB/c and C3H mice; the yellow region is HLA-II exclusive 1, which includes DPB1 and DRB; the blue region is HLA-II exclusive 2, which comprises DQA1/DQB1; and the green region is a cross-reactive cluster (H2 major) with H2-Au, b, k, s, q, and 2 HLA subtypes. The extraction capacity of adjacent MHC molecules was similar.

A heatmap of hierarchical clustering comparing different MHC-II subtypes is shown in **Figure 3**. The 35 MHC-II molecules were divided into four groups, including two cross-reactive and two HLA-II–exclusive clusters. The cross-reactive (HLA major) cluster mainly included DRB1 and three H2 subtypes (H2-Ed, H2-Ek, and H2-Ad) (corresponding to BALB/c and C3H mice). MHC-II in this group had similar immune capacities. HLA-II exclusive 1 comprised DPB1 and DRB and HLA-II exclusive two comprised DQA1/DQB1. The cross-reactive (H2 major) cluster included H2-A (u, b, k, s, and q); these H2 subtypes differed significantly from human HLA-II molecules.

### HLA-II Distribution Unrelated to HTNV GP-Associated Host–Virus Interaction

Allele frequencies were searched in the website (http://www.allelefrequencies.net/hla6006a.asp). The distribution of different alleles in same supertype is highly expressed in different regions and races. In **Figure S3**, there was no significant discrepancy in the ability of different alleles in same supertype to present Hantaan virus peptides. Proved that individuals from different geographic regions and ethnic groups have different HLA-II alleles within the same HLA-II family, exhibiting no significant differences in HTNV GP antigen presentation capacity; thus, geographic stratification has no effect on host–virus interaction.

## Discussion

In this study, the 1,121 15-mer epitopes derived from HTNV GP were analyzed by in silico and experimental approaches. We identified 37 epitopes that cross-reacted with H2 and HLA-II supertypes; moreover, 181 epitopes for H2 and 148 epitopes for different HLA-II supertypes were highly conserved in Orthohantavirus. Of these, the epitopes at a.a. 1–32, 138–189, 208–224, 438–521, 779–814, and 910–941 showed strong binding ability in the different subtypes. Moreover, epitope hotspots overlapped with predicted clusters at a.a. 1–18, 208–210, 138–175, 438–507, 779–800, and 910–927.

Most research to date on viral epitopes has combined bioinformatics-based prediction and molecular dynamic simulations. In our research, we used four in silico tools to estimate the binding strength between HTNV GP 15-mer peptides and MHC-II. Prediction has several advantages. Analysis of epitopes can help to avoid experimental errors, and results obtained by multiple methods are more objective and accurate than those obtained from a single set of experiments or using a single platform. Furthermore, in this study we were able to identify all possible 15-mer peptides of the full-length GP, which would be almost impossible to derive and test experimentally because of cost and time constraints.

The ELISpot assay is widely used to evaluate antigen-specific cytokines and antibody secretion at the single-cell level (Ji and Forsthuber, 2016). Interferon (IFN)-γ is a key factor in cellular immunity, which coordinates numerous protective functions and induces an antiviral state (Kak et al., 2018). We measured IFN-γ level to assess antiviral capacity. Antigen-specific T cells produce multiple cytokines including other Th1 cytokines (eg, interleukin [IL]-2 and tumor necrosis factor [TNF]-α) and Th2 cytokines (eg, IL-15 and IL-13). However, as these T cells represent only a small proportion of the total T cell population, detection of these cytokines by ELISpot has limited utility (Letsch and Scheibenbogen, 2003). On the other hand, ELISpot is more sensitive than other experimental procedures such as intracellular cytokine cytometry (Letsch and Scheibenbogen, 2003) and less complex than *in situ* tetramer staining (Simoni et al., 2019).

The ELISpot assay verified the accuracy of the prediction, and the conservation of the dominant epitopes was also analyzed based on the prediction. Evolutionarily conserved epitopes can provide long-term immunity against viruses regardless of strain variation; epitopes with a high degree of conservation, strong immunogenicity, and non–self-reactivity in humans exhibit good coverage in different populations and are ideal for vaccine development (Maciel et al., 2008; Mirza et al., 2016; Pradhan et al., 2017; Zheng et al., 2018; Ul-Rahman and Shabbir, 2019). Based on these criteria, we got 10 clusters, 6 of 10 epitopes were supported by ELISpot. Thus, our strategy of using multiple prediction tools with ELISpot assay validation has advantages over other approaches (Demharter et al., 2019).

Experiment found that epitope hotspots overlapped with predicted clusters. These hotspots can inform the design of vaccines that promote herd immunity (Harjanto et al., 2014). Herd immunity is the situation where the possibility of an effective contact between an infected and a susceptible person is reduced when adequate numbers of individuals in a group or population have immunity against the pathogen (Smith, 2019). Herd immunity is affected by pathogen structure and immunogenicity and their interaction with the host immune system (Mallory et al., 2018). It has been suggested that the minimum percentage of immune individuals necessary for herd immunity against severe acute respiratory syndrome coronavirus 2 (SARS-CoV-2) is 50% to 66.66%, which is difficult to attain in a short time (Papachristodoulou et al., 2020). Research on cross-protection by vaccines is critical for achieving this end. Dominant epitopes shared by MHC superfamilies can promote the establishment of herd immunity; the predicted and experimentally validated dominant epitopes of MHC-II identified in our study comprised not only single sites (a.a. 44, 113, 335, etc) but also hotspots that met this requirement.

Inoculations with inactivated HTNV virus vaccine have reduced the incidence of HFRS (Yi et al., 2018). However, it is well known that inactivated vaccines do not provide long-lasting immunity without multiple boosters (Song et al., 2016). Therefore, many studies have explored novel candidate vaccines against hantavirus with new epitope designs (Sankar et al., 2017a; Sankar et al., 2017b; Conte et al., 2019) and different methods evaluating efficacy (Zhao et al., 2012; Hooper et al., 2013; Ma et al., 2016). These studies mainly focused on CD8+ T cells and B cell-mediated immunity. It has been demonstrated that immune memory was induced with long-term protection against HTNV by MHC-II (Jiang et al., 2017; Jiang et al., 2018). In the current study, we used different strains of mice injected with vaccine and live virus, and the epitopes confirmed by ELISpot and identified in previous studies (Ma et al., 2015) were compared with results predicted for corresponding MHC subtypes. Using mice with different MHC-II haplotypes allowed us to comprehensively analyze discrepancies between predicted and experimentally determined epitopes. In contrast with vaccines, natural infection caused by live viruses elicits a vigorous T cell response (Avsic-Zupanc et al., 2019), which includes a systemic antiviral immune response and helper T (Th) cell-mediated B cell activation that are important for disease resolution and recovery. Thus, activating the T-cell response is a potential treatment strategy for viral infections (Toor et al., 2021).

T cell receptors (TCRs) not only recognize antigenic peptide but also polymorphisms of MHC during antigen presentation by APCs or MHC-expressing cells, which is known as MHC restriction (Hesnard et al., 2016). Epitopes with cross-reactivity between human and mouse include a wide range of MHC-II molecules of different genera, apparently free of MHC restriction and preferentially presented during the immune response. For instance, the distribution of dominant epitopes in H2d is similar to that in HLA-II superfamilies. While the functional significance of this observation is unclear, it is worth noting that highly similar HTNV Gc epitopes were identified in BALB/c mice and human (Jiang et al., 2018). Thus, in the absence of humanized HLA transgenic mice, the BALB/c strain may be a useful alternative for experiments investigating MHC-II.

Previous studies have evaluated the efficacy of vaccines that cross-react with heterologous hantaviruses and yielded antibodies that were shown to neutralize 3 HFRS hantaviruses (Custer et al., 2003) or 7 HPS and HFRS hantaviruses (Hooper et al., 2006). Conservative epitopes that can elicit an immune response to interspecific viruses can increase the range of protective immunity to include both known and unknown hantaviruses that cause human diseases. It was reported that SARS-CoV-2–cross-reactive memory T cells were present in 28% to 50% of individuals who had not been exposed to SARS-CoV-2 (Lipsitch et al., 2020). Furthermore, homologous sequences from human coronaviruses including SARS-CoV-2 were shown to exhibit immune cross-reactivity, which mainly involved canonical memory CD4+ T cells (Mateus et al., 2020). Our findings increase the understanding of cross-protection mechanisms and provide guidance for the development of vaccines not only for HPS and HFRS, but also for other viral disease including dengue fever (flavivirus), Ebola and Marburg hemorrhagic fever (filovirus), as well as 2019 novel coronavirus diseases (COVID-19) caused by SARS-CoV-2.

## Data Availability Statement

The original contributions presented in the study are included in the article/**Supplementary Material**. Further inquiries can be directed to the corresponding authors.

## Ethics Statement

The animal study was reviewed and approved by experimental animal ethics committee of Fourth Military Medical University.

## Author Contributions

All authors have made substantial contributions to the manuscript. KY, DJ, and HS contributed to the original conception and design of the study. HS, YaL, and JZ predicted epitopes. HS, GX, YF, TW, SX, YuL, BS, and DJ carried out wet-lab experiments. HS, LC, and DJ evaluated experimental results. HS, ZL, and DJ conducted conservative and interactive analysis. LC performed viral challenge assay and relative experiments under BSL-3 conditions. HS, ZL, ML, JX, and DJ presented the tables and figures. HS drafted the manuscript and DJ revised it critically. KY and DJ supervised the whole project. XZ, YS, SY, YunZ, and YusZ assisted the experiments. All authors contributed to the article and approved the submitted version

## Funding

This study was supported by the National Natural Science Foundation of China (No. 81772763 and No. 82073154 to KY) and Key Research and Development Program of Shaanxi Province (No. 2020SF-200 to KY).

## Conflict of Interest

The authors declare that the research was conducted in the absence of any commercial or financial relationships that could be construed as a potential conflict of interest.

## Supplementary Material

Supplementary Figure 1 IFN-γ ELISpot results for epitope validation. “Inact” refers to bivalent HFRS-inactivated vaccine; and “Virus” refers to HTNV strain 76-118. Groups **(A–D)** are the results obtained from C57, BALB/c, C3H, and Kunming mice. Experimentally derived epitopes were screened and compared to predicted epitopes.

Supplementary Figure 2 Conservation analysis by multiple sequence alignment. The overall height of the stack indicates sequence conservation at that position, while the height of symbols within the stack indicates the relative frequency of each a.a. or nucleic acid at that position, with a.a.s colored according to their chemical properties as follows: polar (G, S, T, Y, C, Q, and N), green; basic (K, R, and H), blue; acidic (D and E), red; and hydrophobic (A, V, L, I, P, W, F, and M), black. Predicted epitopes from human, mouse, and both are underlined in blue, red, and orange, respectively.

Supplementary Figure 3 Heatmap illustrating the interaction between MHC-II molecules and predicted epitopes. Columns and rows show MHC-II subtypes and epitopes, respectively. Darker colors represent a stronger interaction (lower %rank).
