# Supplementary figures and images for: Integrative Analysis of HTNV Glycoprotein Derived MHC II Epitopes by In Silico Prediction and Experimental Validation
Source: Front Cell Infect Microbiol. 2021 Jul 19;11:671694. doi: 10.3389/fcimb.2021.671694 (PMC8326763; doi:10.3389/fcimb.2021.671694)

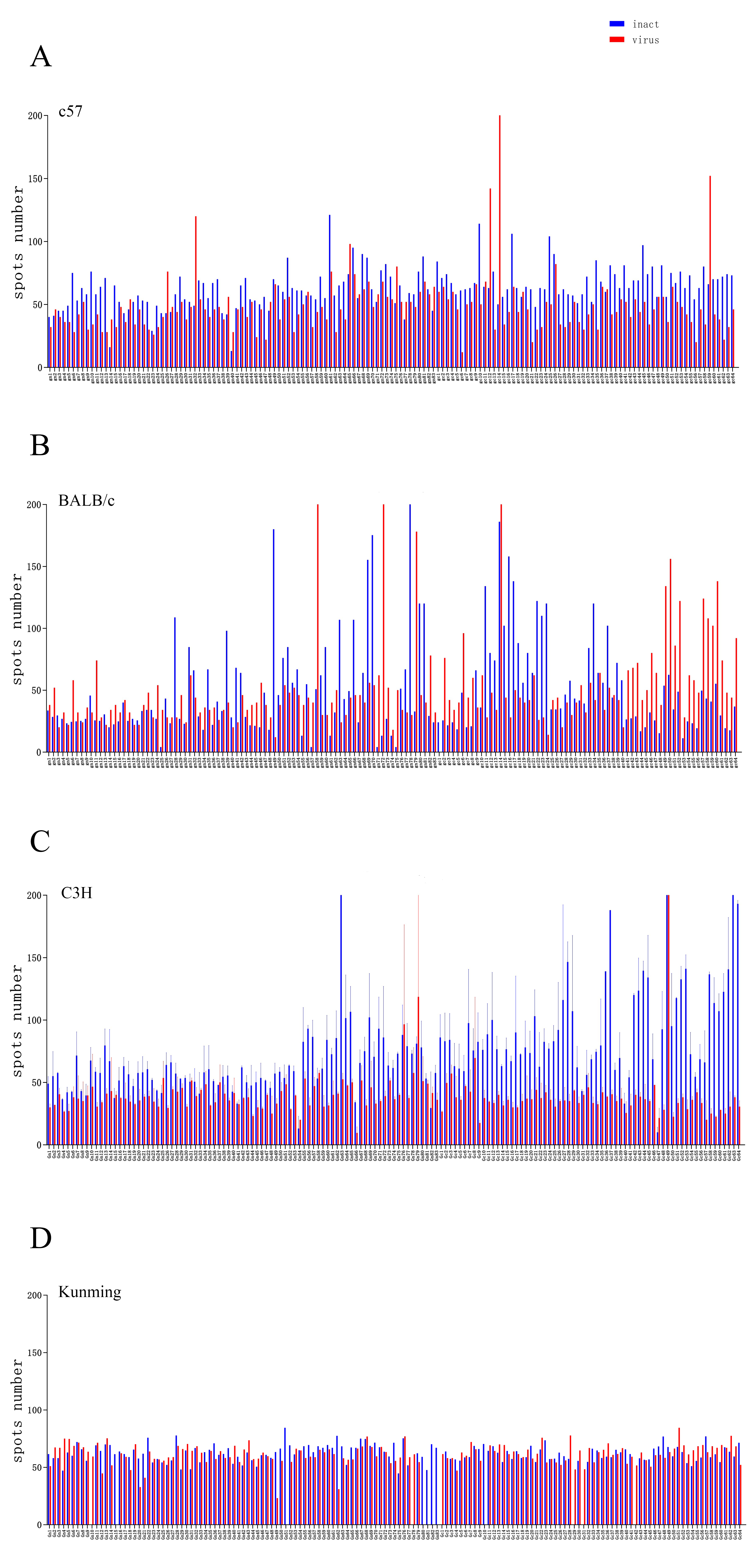

Supplement: Supplementary Figure 1 — IFN-γ ELISpot results for epitope validation. “Inact” refers to bivalent HFRS-inactivated vaccine; and “Virus” refers to HTNV strain 76-118. Groups (A–D) are the results obtained from C57, BALB/c, C3H, and Kunming mice. Experimentally derived epitopes were screened and compared to predicted epitopes. [file Image_1.jpg]

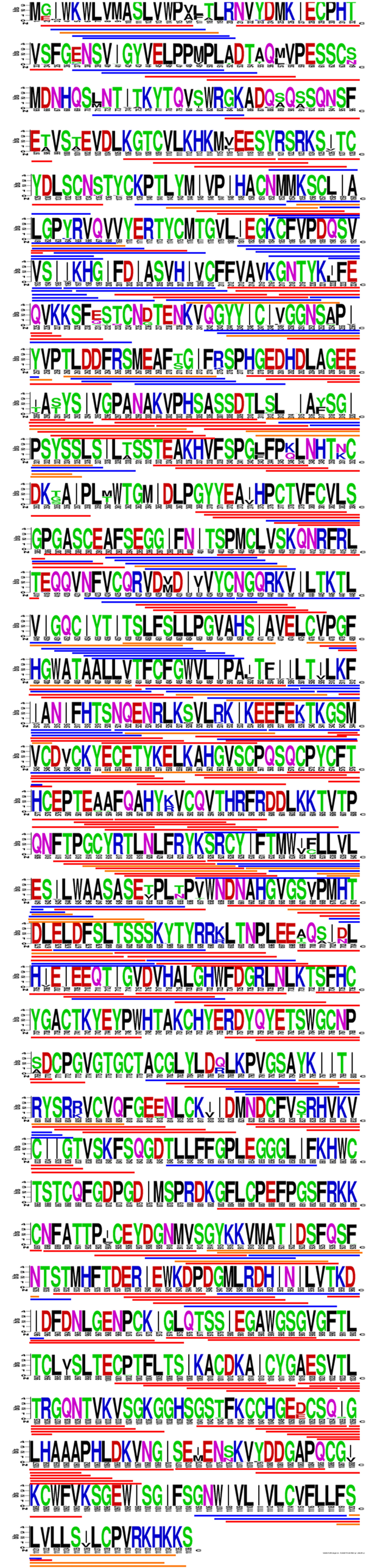

Supplement: Supplementary Figure 2 — Conservation analysis by multiple sequence alignment. The overall height of the stack indicates sequence conservation at that position, while the height of symbols within the stack indicates the relative frequency of each a.a. or nucleic acid at that position, with a.a.s colored according to their chemical properties as follows: polar (G, S, T, Y, C, Q, and N), green; basic (K, R, and H), blue; acidic (D and E), red; and hydrophobic (A, V, L, I, P, W, F, and M), black. Predicted epitopes from human, mouse, and both are underlined in blue, red, and orange, respectively. [file Image_2.jpg]

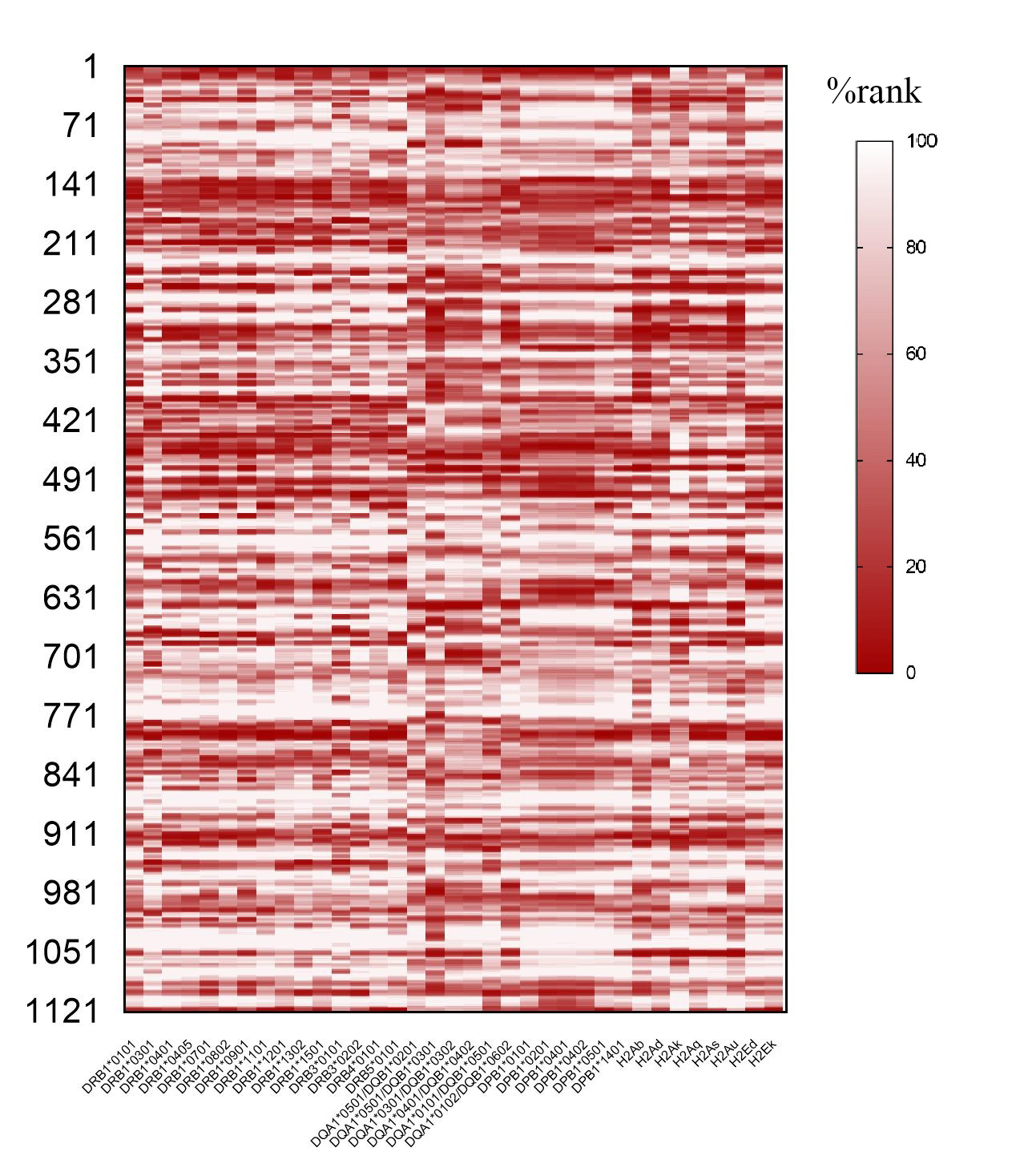

Supplement: Supplementary Figure 3 — Heatmap illustrating the interaction between MHC-II molecules and predicted epitopes. Columns and rows show MHC-II subtypes and epitopes, respectively. Darker colors represent a stronger interaction (lower %rank). [file Image_3.jpg]
